# The Role of Personal and Job Resources in the Relationship between Psychosocial Job Demands, Mental Strain, and Health Problems

**DOI:** 10.3389/fpsyg.2016.01214

**Published:** 2016-08-17

**Authors:** Hannes Mayerl, Erwin Stolz, Anja Waxenegger, Éva Rásky, Wolfgang Freidl

**Affiliations:** Institute of Social Medicine and Epidemiology, Medical University of GrazGraz, Austria

**Keywords:** job demands-resources model, job resources, personal resources, mental strain, health problems

## Abstract

Recent research highlights the importance of both job resources and personal resources in the job demands-resources model. However, the results of previous studies on how these resources are related to each other and how they operate in relation to the health-impairment process of the job demands-resources model are ambiguous. Thus, the authors tested an alternative model, considering job and personal resources to be domains of the same underlying factor and linking this factor to the health-impairment process. Survey data of two Austrian occupational samples (*N*_1_ = 8657 and *N*_2_ = 9536) were analyzed using confirmatory factor analysis (CFA) and structural equation modeling (SEM). The results revealed that job and personal resources can be considered as indicators of a single resources factor which was negatively related to psychosocial job demands, mental strain, and health problems. Confirming previous studies, we further found that mental strain mediated the relationship between psychosocial job demands and health problems. Our findings suggest that interventions aimed at maintaining health in the context of work may take action on three levels: (1) the prevention of extensive job demands, (2) the reduction of work-related mental strain, and (3) the strengthening of resources.

## 1. Introduction

There is good empirical evidence for the adverse effects of psychosocial stress at work on mental and somatic health (Siegrist, 2008; Leka and Jain, 2010; Nixon et al., 2011). Furthermore, a recent systematic review has shown that the economic crisis with its peak in 2009 went along with staff reduction, increased workload, or a rise in unemployment, all of which resulted in an increased rate of health problems (Mucci et al., 2016). Since the economic crisis is still on-going in a number of countries today and occupational demands seem to be on the rise (Leka and Jain, 2010), occupational stress research should further examine valuable factors that have the potential to attenuate the negative impact of adverse working conditions on health.

A number of work-related stress models have been proposed in order to explain how certain work characteristics affect mental and somatic health (Mark and Smith, 2008). One of them is the job demands-control model (JD-C model; Karasek, 1979; Karasek and Theorell, 1990). According to this model, stress reactions are due to a combination of high demands and low decision latitude or control at work. Another frequently cited model is the effort-reward model (ER model), which claims that an imbalance between (high) efforts and (low) rewards results in sustained stress reactions (Siegrist, 1996).

One prominent model integrating previous concepts is the job demands-resources (JD-R) model (Demerouti et al., 2001). Originally, the JD-R model considered two factors: job demands and job resources. Job demands were defined as “those physical, psychological, social, or organizational aspects of the job that require sustained physical and/or psychological (cognitive and emotional) effort and are therefore associated with certain physiological and/or psychological costs” (Bakker et al., 2003, p. 344). Job resources, in contrast, were related to job factors (1) that attenuate job demands and the related costs, (2) facilitate goal achievement, and (3) are beneficial for personal growth and development (Demerouti et al., 2001; Bakker et al., 2003). The JD-R model predicts health problems as a result of high job demands and poor job resources (Bakker et al., 2005). However, there is not the one and only JD-R model on which all previous studies were based. Instead, the JD-R model represents a heuristic way of thinking, which rather than restricting itself to a specific and well-defined set of job characteristics, allows the inclusion of any demands and resources in the model (Schaufeli and Taris, 2014, p. 44). Moreover, since its first publication in 2001, several revisions and extensions have been suggested and tested (for a review see Schaufeli and Taris, 2014).

One of these extensions concerned the inclusion of mental strain as a mediator in the relationship between job demands and health (Schaufeli and Bakker, 2004). The psychological process underlying this relationship was referred to as energetic or health-impairment process (as opposed to the motivational process) and justified by Hockey's (1997) state regulation model of compensatory control. According to this model, individuals strive to maintain performance levels in order to achieve the defined objectives. Especially in highly demanding situations, this requires an investment of mental effort. Although such an investment leads to mental energy loss, health consequences are minor when the magnitude of energy utilized remains within available reserve limits. However, where demands exceed the individual energy budget, it is likely that efforts are further increased to a level beyond the individual's reserve capacities. Such a strategy to cope with extensive demands has unwanted side-effects that are manifested as mental strain and physiological reactions. In this context, mental strain refers to negative mood states and feelings of mental impairment such as anxiety, irritability, exhaustion, or cynicism (Hockey, 1997; Bakker et al., 2003). Physiological reactions involve typical stress responses such as sympathetic nervous system activation or neuroendocrine responses. In the long term, these side-effects can have detrimental effects on the body (see also McEwen, 1998).

Investigating this health-impairment process, research has shown that mental strain (operationalized as core dimensions of burnout: exhaustion and cynicism; Maslach et al., 2001) mediated the relationship between job demands and health (Schaufeli and Bakker, 2004; Hakanen et al., 2006). High job demands were related to higher levels of mental strain which in turn were associated with ill health. This indirect pathway fully explained the job demands-health link, indicating that mental strain entirely accounts for the effect of job demands on health. These findings were, however, countered by a recent study, also demonstrating the existence of a direct relationship between job demands and health (Korunka et al., 2009).

A potential weakness of most previous research on the JD-R model is that only valuable factors in relation to work were considered, while neglecting individual characteristics that might be beneficial in a broad array of contexts. As Kalimo et al. (2002) have shown, both job resources and individual characteristics are good long-term predictors of psychological and physiological stress reactions. Hence, recent research on the JD-R model has come to also involve personal resources, often defined as factors of the self generally associated with resilience (Hobfoll et al., 2003). Especially self-efficacy, self-esteem, or optimism have been studied in this context (Xanthopoulou et al., 2009a,b; Airila et al., 2014). Although personal resources should, by definition, have the potential to attenuate the negative impact of job demands on health outcomes and/or well-being, this assumption was not confirmed in a recent study. Instead of revealing the personal resources as a moderator in the link between job demands and exhaustion, they were found to mediate the relationship between the job resources and exhaustion (Xanthopoulou et al., 2007). This finding was justified by conservation of resources (COR) theory (Hobfoll, 1989) which, on the one hand, states that individuals strive to preserve, protect, and enlarge their resources, and on the other hand, that different types of resources do not exist in isolation, but rather build resource caravans in that resources tend to generate other resources (Hobfoll, 2002). Thus, job resources can be seen as a hypothetical antecedent of personal resources insofar as a resourceful work environment facilitates the accumulation of personal resources such as self-esteem or self-efficacy. As Xanthopoulou et al. (2007) have shown, the reversed direction may also be reasonable as job resources may be seen as an antecedent of personal resources. That is, individuals high in personal resources may be more able to also create a resourceful work environment. Thinking one step further, the relationship between personal and job resources can also be seen as a dynamic relationship acting in cycles (see also Kohn and Schooler, 1982). Indeed, a longitudinal study demonstrated that personal resources and job resources were mutually related to each other (Xanthopoulou et al., 2009a).

Instead of considering job resources and personal resources as two separate (albeit interrelated) constructs, an alternative point of view would be to see them as facets of a common underlying factor. In the study of Xanthopoulou et al. (2007) all indicators of job and personal resources were positively related to each other, to a moderately high extent. Employees who perceived high job resources also had more positive beliefs about themselves and their abilities. These findings are not at all surprising, since personal resources and job resources overlap to a considerable extent. For example, a person who evaluates his or her own skills as sufficient to cope with a variety of challenges (i.e., having more personal resources) will be more likely to view work as controllable and therefore report more autonomy and decision latitude at the workplace (i.e., having more job resources at their disposal). The positive correlations between different kinds of personal resources and job resources may be held to reflect the existence of a common core or, in other words, different types of resources can be considered as indicators of one and the same underlying factor. However, it seems important to say that such an underlying resources factor should not be considered as a real thing, instead of a hypothetical concept, or that it corresponds to a single domain that can be easily measured (Kline, 2011, p. 231). Instead, the resources factor is seen as a multifaceted hypothetical construct, characterized by domains of both job and personal resources. This reasoning is in line with other theories regarding the hierarchical structuring of psychological concepts. One prominent example is Gardner's (2006) theory of intelligence, in which “intelligence” is considered as a multidimensional concept that comprises a set of different domains of intelligence, such as logical-mathematical, linguistic, or interpersonal domains. To our knowledge, however, previous research has never attempted to model a multidimensional resources factor, composed of both job and personal aspects.

The relationship between job and personal resources may also depend on how personal resources are defined and operationalized. Studies in this context relate personal resources only to cognitive aspects associated with resilience (Xanthopoulou et al., 2009a,b; Airila et al., 2014). This may be a limitation since physical and social aspects were also found to be important. For instance, Ensel and Lin (2004) have shown that the better the physical fitness of a person was, the less physical and psychological distress (such as somatic complaints and depressive symptoms) were reported. In their study, physical fitness was also related to resources of the self, i.e., persons high in physical fitness also showed higher levels of self-esteem. This further underlines the assumption that different kinds of resources are interrelated and that they may have a common core. In addition, social support was also found to be a crucial factor in the link between job demands and health outcomes. A meta-analysis demonstrated high levels of social support to be associated with less experience of work-related strain and less workplace stressors perceived (Viswesvaran et al., 1999). On the basis of these findings, we defined personal resources more broadly, and in line with a biopsychosocial way of thinking (Engel, 1977), as those physical, mental, and social characteristics of an individual which strengthen the resilience against several kinds of challenges, not only related to the work environment, but rather to a broad array of contexts.

To summarize, we found good empirical support for the health-impairment process of the JD-R model. However, the role of personal and job resources in the health-impairment process needs to be further examined. The main objective of the current study was to define an alternative model that combines both job and personal resources to a common resources factor and to link this factor to the health-impairment process of the JD-R model.

**Figure 1:**
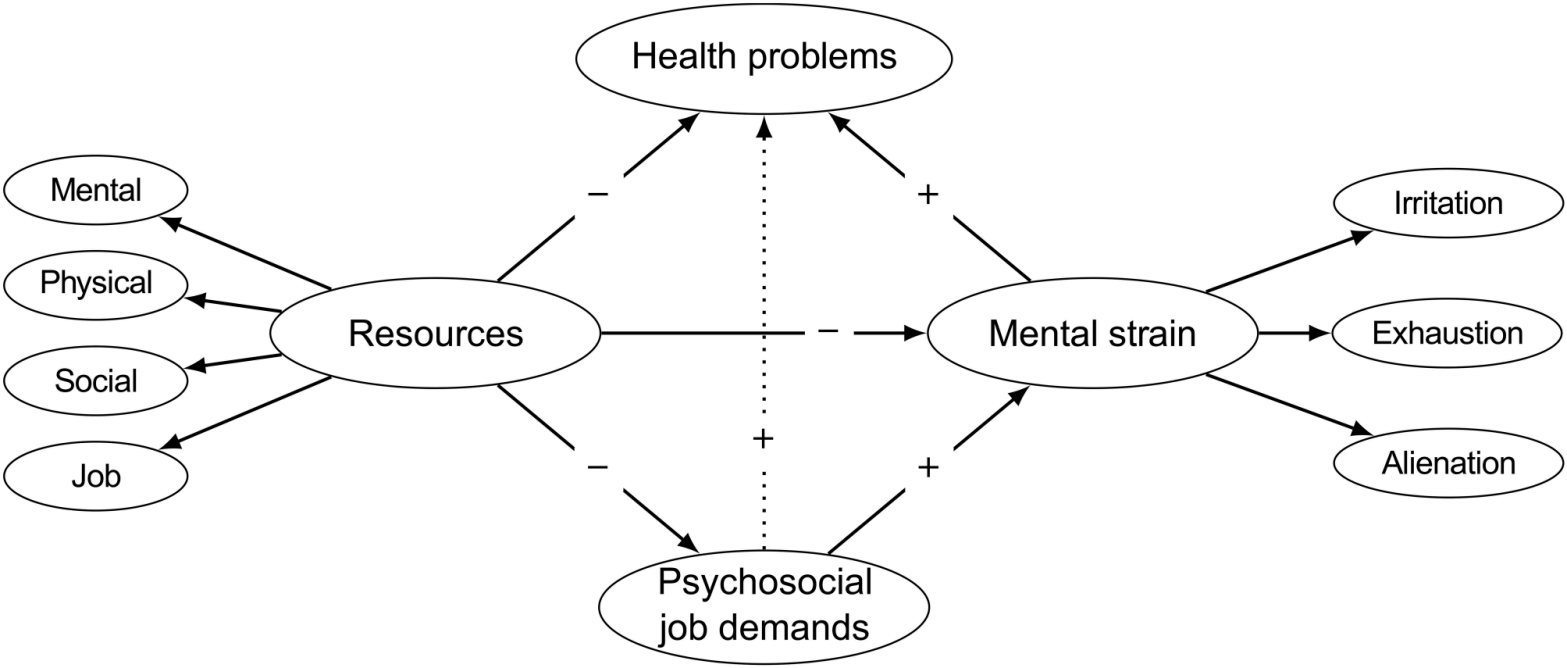
**Hypothesized relationships of the research model**. “+” indicates a predicted positive relationship and “−” a predicted negative association.

The assumptions of our hypothesized model are illustrated in Figure 1. Our first hypothesis was that beneficial work characteristics and valuable aspects of an individual are both indicators of a common underlying factor. Different types of job and personal resources can therefore be combined to form a single resources factor. In our study, we operationalized job resources by the amount of autonomy/decision latitude at work (Karasek, 1979) and by the amount of satisfaction with rewards (Siegrist, 1996). To account for the physical, mental, and social dimensions of personal resources we considered three indicators: (1) self-rated physical fitness, (2) generalized self-efficacy, defined as a global belief of being able to cope with a wide range of challenging situations (Schwarzer et al., 1997), and (3) subjectively perceived social support outside of work.

Furthermore, based on previous research findings on the health-impairment process of the JD-R model, we assumed that job demands exert their influence on health via an indirect pathway through mental strain. Job demands were operationalized as the burden emerging from psychosocial demands at work, including both psychological and social aspects of the job that require sustained efforts on the part of workers (Demerouti et al., 2001). As an indicator for health problems, we used the participants' self-assessed degree of suffering from somatic and psychosomatic complaints. Regarding mental strain, we considered three psychological responses associated with extensive demands in the context of work, namely irritation, exhaustion, and alienation. For this purpose irritation referred to a state of mental impairment including cognitive (rumination) and emotional irritation (irritability) (Mohr et al., 2006). Exhaustion represented feelings of being overtaxed and depleted of one's resources due to overwhelming job demands (Maslach et al., 2001), while alienation referred to a cognitive state of separation or estrangement from the self (Fromm, 1955; Kanungo, 1979). This operationalization of mental strain allowed us to generalize previous findings beyond burnout. We hypothesized that the more psychosocial job demands were perceived, the greater the level of mental strain would be, which in turn would be related to more health problems. Since previous research findings are inconsistent regarding the question as to whether the relationship between job demands and health is completely mediated by mental strain or only partially, we further examined the direct pathway from psychosocial job demands to health problems (see the dotted line in Figure 1). If it exists at all, this link is expected to be a positive one, in that high psychosocial job demands would be associated with a greater amount of health problems.

The resources factor was hypothesized to be negatively related to psychosocial job demands, mental strain, and health problems. More specifically, we expected the perceived burden due to psychosocial job demands to depend (1) on how participants evaluated their own abilities and capacities (Lazarus and Folkman, 1984), and (2) on the degree of control at work (Karasek, 1979) and the belief of being sufficiently rewarded for one's efforts (Siegrist, 1996). An individual high in these personal and job resources will be less likely to evaluate the working environment as a burden than an individual low in such resources. Thus, we hypothesized that: the more resources available, the lower the perceived burden due to psychosocial job demands. Previous research also showed personal and job resources to be good predictors of somatic and mental health status. For example, personal resources, such as sense of coherence or affect balance, were found to be associated with higher psychological and physical quality of life and with less somatic problems (Freidl et al., 1999). Similarly, an increase in job resources (such as job control) lowered the risk for coronary heart disease, absenteeism, and depression (Karasek, 1990). We correspondingly hypothesized that persons high in resources would report lower levels of mental strain and of health problems.

## 2. Methods

### 2.1. Data

On behalf of the *Arbeiterkammer Oberösterreich*, the *Institute for Empirical Social Studies (IFES)* collected data among the Austrian working population using proportionally stratified random sampling. Participants were either employed or self-employed. Trained interviewers conducted personal face-to-face interviews to gather self-reported data concerning 26 subject areas (e.g., demographics, working conditions, mental, and somatic health). Questioning was structured and standardized, while most of the questions had a limited set of response categories. Each participant had the right to refuse participation at any point of the interview.

We processed the data from two samples: while data from the first sample were used to investigate and refine the hypothesized measurement models, those of the second sample served to test the research model as a whole. The samples were separated according to the date of the interview. The first sample was surveyed between 2009 and 2011, and the second sample between 2012 and 2014. The reason for using this criterion instead of randomly splitting the data set was the requirement to test the research model on the latest available data.

The first sample included *N* = 8657 participants, with a mean age of 39.4 years (*SD* = 11.2; range: 15–75 years). Four thousand two hundred and seventeen participants were male (49%) and 4440 (51%) female. A rate of 9.4% had at most completed compulsory school, 62.8% had graduated from vocational school or were skilled workers with an apprenticeship certificate, 15.0% held a high school diploma, and 12.8% a university degree. There was only a small amount of missing values (0.67%).

The second sample consisted of *N* = 9536 participants and the mean age was 39.8 years (*SD* = 11.8; range: 15–85 years). Four thousand eight hundred and twenty-five participants were male (51%) and 4711 female (49%). A rate of 9.1% had completed compulsory school, 64.5% had graduated from vocational school or were skilled workers with an apprenticeship certificate, 13.4% held a high school diploma, and 13.0% a university degree. 0.69% of the data were missing.

### 2.2. Ethics approval

Research was carried out in compliance with the principles defined in the Helsinki Declaration. The interviewers informed the participants about the study objectives and on confidentiality and anonymity. Interviews were conducted after verbal informed consent. In case of 15–17 year-olds (in total 0.58%), both the minor participant and a legal guardian had to agree. The Ethics Committee of the Medical University of Graz approved the conductance of the current study (EK-number: 27–251 ex 14/15; date: 24.04.2015).

### 2.3. Measures

#### 2.3.1. Psychosocial job demands (JD_1_–JD_8_)

Psychosocial job demands referred to those psychological and social aspects at the workplace that were subjectively perceived as a burden. Participants were asked to indicate, on a 5-point scale (“1 = not stressed” to “5 = strongly stressed”), how much they felt burdened by time pressure (JD_1_), high responsibility (JD_2_), emotionally burdening and annoying work (JD_3_), excessive working hours (JD_4_), irregular working hours (JD_5_), permanent monitoring (JD_6_), lack of support from colleagues (JD_7_), and lack of support from the supervisor (JD_8_).

#### 2.3.2. Mental strain

Mental strain consisted of the constructs irritation, exhaustion, and alienation. (1) Irritation was assessed using a modified version of the German Irritation Scale of Mohr et al. (2005). The original scale has a two-dimensional factor structure (namely cognitive and emotional irritation) and was developed for stress research and the assessment of psychological strain associated with the working environment. The modified version of this scale accounts for the emotional dimension (irritability), with three items (e.g., “I anger quickly.”), and the cognitive dimension (rumination), with two items (e.g., “I have difficulties to unwind after work.”). (2) Alienation was assessed with three items, based on a subscale measuring the subjective feeling of being estranged from the self (e.g., “I often do not understand what is actually happening.”) (Freidl, 1997). (3) Exhaustion was measured using three items representing feelings of being overextended and exhausted from work (e.g., “I feel exhausted due to work.”) (Maslach et al., 2001). All items had to be rated on a 5-point scale (“1 = I do not agree” to “5 = I strongly agree”).

#### 2.3.3. Health problems (HP_1_–HP_11_)

Health problems were measured using 11 items referring to complaints regarding the digestive system, the circulatory system, the musculoskeletal system, the respiratory system, the skin tissue, and psychosomatic issues. Participants rated, on a 5-point scale, how often in the last weeks (“1 = never” to “5 = very often”) they had suffered from: digestive problems (HP_1_), stomach troubles (HP_2_), headaches/migraine (HP_3_), difficulties falling asleep/difficulties remaining asleep (HP_4_), weakness of memory/lack of concentration (HP_5_), muscle tenseness in neck and shoulder regions (HP_6_), back pain (HP_7_), hypertension (HP_8_), palpitations/tachycardia/feeling of pressure on the chest (HP_9_), skin rash/itching/skin redness (HP_10_), and respiratory problems/shortness of breath/breathlessness/asthma (HP_11_).

#### 2.3.4. Personal resources and job resources

Personal resources were comprised of three dimensions: (1) Physical: The physical component was addressed using three items measuring participants' physical constitution on a 5-point rating scale ranging from “1 = very poor” to “5 = very good” (e.g., “How would you assess your physical fitness?”). (2) Mental: Regarding the mental component we referred to the concept of generalized self-efficacy. We applied three items from a German version of the “Generalized Self-efficacy Scale” (e.g., “I can always manage to solve difficult problems if I try hard enough.”) (Schwarzer and Jerusalem, 1995, 1999). Response categories ranged from “1 = I do not agree” to “5 = I strongly agree.” (3) Social: We operationalized the social resource by social support from outside the closer family circle using three items ranging from “1 = do not agree” to “5 = strongly agree” (e.g., “I have persons beyond my immediate family circle, on whom I can count in case of emergency.”).

To assess job resources we selected six items in total, measuring the level of satisfaction with income (JR_1_), working rights (JR_2_), career and development opportunities (JR_3_), occupational training opportunities (JR_4_), opportunity for co-determination at workplace (JR_5_), and decision latitude (JR_6_). Response categories ranged from “1 = not at all satisfied” to “5 = very satisfied.” These items were selected in line with the JD-C model (Karasek, 1979) and the ER model (Siegrist, 1996). Since the original scales of these theories were not available, we considered these items as proxy measures reflecting decision latitude/autonomy at work and occupational rewards (for a similar approach see de Jonge et al., 2000).

### 2.4. Statistical analyses

Statistical analyses were performed with R 3.1.2 (R Core Team, 2014) and R-package lavaan 0.5–17 (Rosseel, 2012). In a first step, we tested the hypothesized measurement models using CFA, and in a second step, we investigated the hypothesized relationships among the constructs using SEM. Since it is often not possible to fully specify a model a priori, the first step usually requires the use of specification searches in order to improve the fit of the model with the data (Anderson and Gerbing, 1988; MacCallum et al., 1992; Raykov and Marcoulides, 2012, p. 49). We used modification indexes as a diagnostic tool to detect and correct specification errors in an initially defined model. However, such a purely data-driven approach (by evaluating and modifying hierarchical models using the same data) would be susceptible to capitalization on chance (MacCallum et al., 1992). Hence, we only applied modifications that were theoretically justifiable. Furthermore, we tested whether the redefined models could be generalized to a second sample. That is, we used the data of the first sample to investigate and modify the measurement models and the data of the second sample in order to validate our findings and test the research model as a whole. As stated by Anderson and Gerbing (1988), the separate examination (and modification) of the measurement models prior to the estimation of the entire model (measurement and structural parts) permits a more comprehensive evaluation of construct validity. Moreover, we tested our research model against an alternative (nested) model (in order to find out whether mental strain fully, or only partially, mediates the relationship between job demands and health problems). For this model trimming procedure (Kline, 2011, p. 214), we used the scaled χ^2^-difference test (Satorra and Bentler, 2001, 2010) and examined the decline in fit indexes (for similar approaches used in the context of the JD-R model, see Schaufeli and Bakker, 2004; Hakanen et al., 2006; Korunka et al., 2009).

To account for the ordinal character of the data in CFA and SEM, we relied on polychoric correlations and diagonally weighted least squares estimation with robust standard errors and mean- and variance-adjusted test statistics (WLSMV estimation) (see Muthén, 1984; Jöreskog, 1994). Pairwise deletion was used to handle the missing data. Model fit was assessed using the χ^2^-statistic, the Tucker-Lewis-Index (*TLI*), the Comparative-Fit-Index (*CFI*), and the Root Mean Square Error of Approximation (*RMSEA*) with 90%-confidence intervals. In evaluating model fit, we concentrated on the *CFI*, the *TLI* and the *RMSEA*, since these indexes where found to be less sensitive to sample size in comparison to the χ^2^-test (Brannick, 1995; Cheung and Rensvold, 2002) and to other widely used indexes such as the goodness-of-fit statistic (Marsh et al., 1988; Sharma et al., 2005; Iacobucci, 2010). A *TLI* and a *CFI* of ≥0.95 and a *RMSEA* of ≤ 0.06 are indicative of a good model fit (Hu and Bentler, 1999).

To assess composite reliabilities of the scales, we computed coefficient ω using R-package semTools 0.4–9 (Pornprasertmanit et al., 2014). This made it possible to evaluate model-based reliability by accounting for correlated measurement errors (Raykov, 2001).

## 3. Results

### 3.1. Measurement models

#### 3.1.1. Psychosocial job demands

As for psychosocial job demands, we tested a one-factor model with the eight aforementioned items as indicators. By examining the modification indices we found to improve model fit by allowing the residual variances of items JD_1_, JD_2_, and JD_3_, the items JD_4_ and JD_5_, and the items JD_6_, JD_7_, and JD_8_ to covary among themselves, respectively. This model showed reasonable fit, χ^2^_(13)_ = 279.43; *CFI* = 0.995; *TLI* = 0.989; *RMSEA* = 0.049, *CI* [0.044, 0.054], and the composite reliability was acceptable, ω = 0.74.

#### 3.1.2. Mental strain

As for mental strain, we formed a second-order factor model with the three latent first-order indicators (i.e., irritation, exhaustion, and alienation). The irritation scale had five items as indicators and the exhaustion and alienation scales each had three item indicators. We a priori decided to let the residual variances of the irritation items covary within each of the two dimensions. The fit of this model was satisfactory, χ^2^_(37)_ = 921.94; *CFI* = 0.997; *TLI* = 0.995; *RMSEA* = 0.053, *CI* [0.050, 0.056]. The reliability of the second-order factor was ω_1_ = 0.76 on level 1 and ω_2_ = 0.82 on level 2. These values indicate the proportion of the second-order factor explaining the total score (level 1) and the variance at first-order factor level (level 2).

#### 3.1.3. Health problems

To assess health problems, we tested a single-factor model with 11 items as indicators. It was found to be reasonable to include the residual covariances of items HP_1_ and HP_2_, and items HP_3_, HP_6_, and HP_7_, respectively. This model showed good fit, χ^2^_(40)_ = 645.18; *CFI* = 0.983; *TLI* = 0.977; *RMSEA* = 0.042, *CI* [0.039, 0.045], and the reliability of this scale was also satisfactory, ω = 0.82.

#### 3.1.4. Resources

As for resources, we tested a second-order model based on the four latent first-order indicators, namely physical, mental, social, and job resources. Among the job resources we let the residual variances of items JR_1_ and JR_2_, items JR_3_ and JR_4_, and items JR_5_ and JR_6_ covary among themselves, respectively. This second-order model fitted well with the data, χ^2^_(83)_ = 1008.40; *CFI* = 0.996; *TLI* = 0.995; *RMSEA* = 0.036, *CI* [0.034, 0.038]. The composite reliability of the second-order factor on levels 1 and 2 was acceptable, ω_1_ = 0.69 and ω_2_ = 0.72. The model-implied correlation between the factor job resources and the factors of physical, mental and social resources were 0.49, 0.46, and 0.37, respectively.

To sum up, all our redefined measurement models showed satisfactory fit and acceptable reliability. Respecifications exclusively concerned the inclusion of residual covariances. All these modifications seemed to be theoretically justifiable. For instance, it seems reasonable to assume an unexplained covariance between items measuring the frequency of digestive problems (HP_1_) and stomach troubles (HP_2_), or between items assessing the burden of excessive (JD_4_) and irregular working hours (JD_5_), which cannot only be accounted for by the underlying factors.

### 3.2. Structural equation modeling

In a next step, we validated the modified measurement models and tested the entire model as summarized in Figure 1 (including the direct pathway from psychosocial job demands to health problems) by means of the second sample. Descriptive statistics, reliabilities, and the correlation matrix for all latent variables in this model are presented in Table 1. All relationships pointed in the expected direction and the reliabilities were satisfactory.

**Table 1:** **Descriptive statistics and the model-implied correlation matrix of the latent variables**.

	***M***	***SD***	**1**	**2**	**2a**	**2b**	**2c**	**3**	**4**	**4a**	**4b**	**4c**	**4d**
1 Psychosocial job demands	1.80	0.74	(0.76)										
2 Mental Strain	1.86	0.79	0.91	(0.87)									
2a Irritation	1.76	0.84	0.83	0.92	(0.78)								
2b Alienation	1.79	1.02	0.63	0.70	0.64	(0.91)							
2c Exhaustion	2.02	1.00	0.80	0.88	0.81	0.62	(0.84)						
3 Health problems	1.50	0.51	0.57	0.62	0.57	0.43	0.55	(0.85)					
4 Resources	3.99	0.54	−0.68	−0.73	−0.67	−0.51	−0.64	−0.62	(0.75)				
4a mental	3.84	0.79	−0.44	−0.47	−0.43	−0.33	−0.42	−0.40	0.65	(0.83)			
4b physical	4.12	0.64	−0.52	−0.56	−0.51	−0.39	−0.49	−0.47	0.77	0.50	(0.83)		
4c social	4.33	0.85	−0.34	−0.36	−0.33	−0.25	−0.32	−0.31	0.50	0.32	0.38	(0.89)	
4d job	3.69	0.76	−0.51	−0.54	−0.50	−0.38	−0.48	−0.46	0.74	0.48	0.57	0.37	(0.79)

*In the diagonal, we report coefficient ω as a measure of reliability. M = mean and SD = standard deviation. The correlation matrix and descriptive statistics for the observed variables can be found in the attached Supplementary Material*.

This model fitted well with the data, χ^2^_(914)_ = 17970.88; *CFI* = 0.966; *TLI* = 0.963; *RMSEA* = 0.044, *CI* [0.044, 0.045]. We further investigated whether the exclusion of the direct pathway from psychosocial job demands to health problems resulted in a considerable decline in model fit, in comparison to the model that included this path. Although the effect of this direct pathway was significant with a standardized coefficient of β = −0.18 (unstandardized *b* = −0.18, *SE* = 0.06, *p* = 0.001), we eliminated this direct pathway since the scaled χ^2^-difference test was not significant at level α = 1% [Δχ^2^_(1)_ = 3.81, *p* = 0.051], and the approximate fit indexes did not diminish (Δ*CFI*, Δ*TLI*, and Δ*RMSEA* < 0.001, respectively).

**Figure 2:**
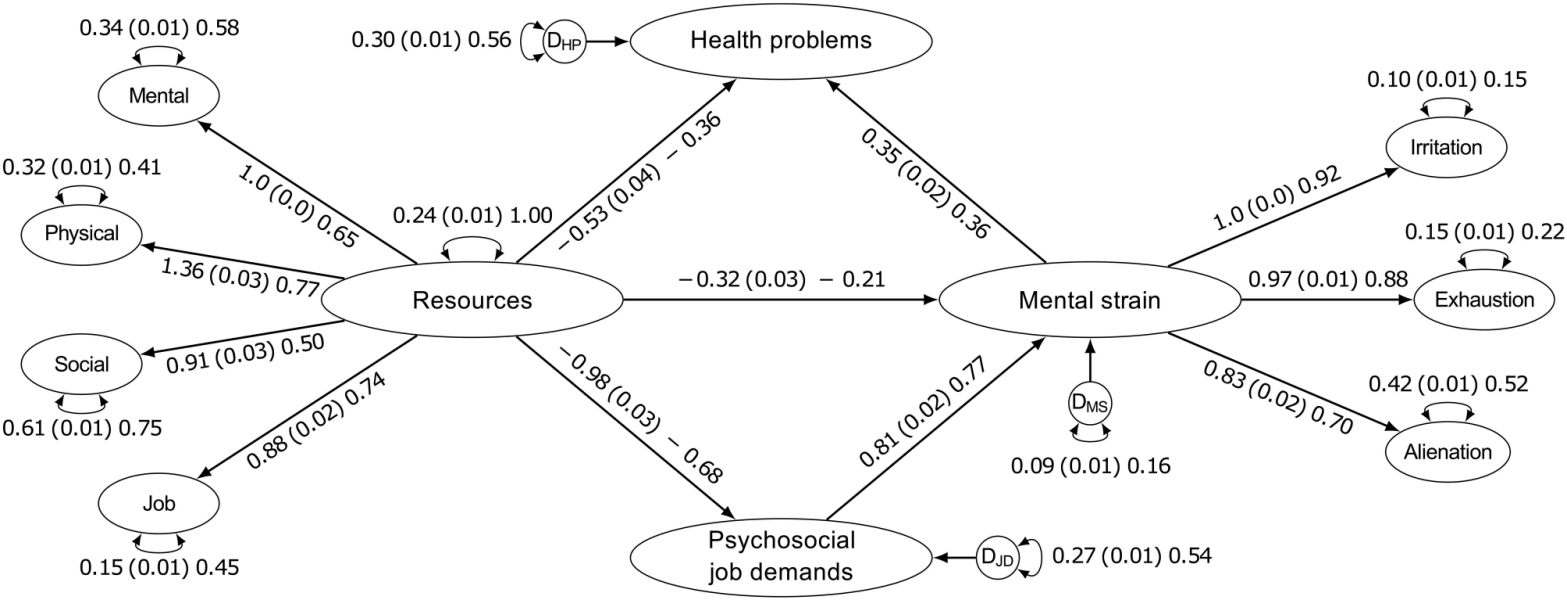
**Results of the final model**. We report the estimates as “unstandardized (standard error) standardized.” D_JD_, D_MS_, and D_HP_ stand for the disturbance variances of the respective endogenous variables. All reported coefficients and factor loadings were significant (all *p*s < 0.001). Due to the high number of observed variables used in this research (45 items in total), we only reported the latent variable model. Model fit of the final model was good: χ^2^_(915)_ = 17833.77; *CFI* = 0.966; *TLI* = 0.964; *RMSEA* = 0.044, 90% *CI* [0.043, 0.045].

The estimates of our final model can be found in Figure 2. All hypothesized relationships pointed in the expected direction. Greater burden due to psychosocial job demands was related to higher levels of mental strain, which in turn was associated with more health problems. This indirect effect was significant (*delta method*) with a standardized coefficient of β = 0.28 (unstandardized *b* = 0.29, *SE* = 0.02, *p* < 0.001). The resources factor was inversely related to psychosocial job demands, mental strain, and health problems, respectively. That is, persons who ranked high in this factor reported a lesser burden due to psychosocial job demands, lower levels of mental strain, and less health problems, respectively.

Moreover, we examined whether the final model held across gender, age groups, and occupational groups. On the one hand, we wanted to test the robustness of the final model among these groups, and on the other hand, we tried to rule out possible confounding. To achieve this, we used a multiple-group approach to test invariance regarding measurement and the structural paths of the final model between (1) men (*N* = 4825) and women (*N* = 4711), (2) the three age groups: young (15–29 years, *N* = 2245), middle (30–50 years, *N* = 5282), and old (51–85 years, *N* = 2009), and (3) the two occupational groups: blue collar worker (*N* = 2529) and white collar worker (98.5%)/entrepreneur (0.3%)/liberal profession (1.2%) (*N* = 7007). We applied a sequential approach by gradually imposing cross-group equality constraints. First, only the same factor structure was imposed on the groups (configural invariance). Then, in a second, third, and fourth step, we additionally constrained the factor loadings (weak invariance), the thresholds (strong invariance), and the residual variances/covariances (strict invariance) to be equal across groups, respectively. The invariance hypothesis of each step was retained if the fit of a more restrictive model did not worsen significantly compared to a lesser restrictive model, according to the scaled χ^2^-difference test (Satorra and Bentler, 2010).

The configural invariance models for gender, χ^2^_(1830)_ = 18667.80; *CFI* = 0.966; *TLI* = 0.963; *RMSEA* = 0.044, *CI* [0.043, 0.045], age, χ^2^_(2745)_ = 18017.98; *CFI* = 0.968; *TLI* = 0.965; *RMSEA* = 0.042, *CI* [0.041, 0.042], and occupational level, χ^2^_(1830)_ = 18294.27; *CFI* = 0.966; *TLI* = 0.963; *RMSEA* = 0.043, *CI* [0.043, 0.044] showed good fit. By constraining both the factor loadings and thresholds, model fit did not decline significantly in any of the three multiple-group models. However, model fit significantly diminished in the fourth step, indicating that the residual variances/covariances differ across groups. Furthermore, we tested whether the structural paths are invariant by additionally constraining the path coefficients of the strong invariance model to be equal across groups. The fit of this model was not significantly worse than that of the strong invariance model in any of the three multiple-group models.

To sum up, the measurement model showed strong invariance across gender, age, and occupational levels, indicating that the observed scores of the constructs have the same meaning among these specific groups. Moreover, also the structural paths were found to be invariant, thereby underscoring the robustness of the model and ruling out possible confounding.

## 4. Discussion

The main objective of the present study was to test an alternative model in order to explain the health-impairment process of the job demands-resources model by integrating job and personal resources. The proposed model fitted well with the data and allowed a good prediction of the burden due to psychosocial job demands, the level of mental strain, and the amount of health problems reported.

More specifically, we found that job resources as well as the physical, mental, and social domains of personal resources can be considered as indicators of the same underlying factor. This confirmed our assumption that different types of resources are interrelated and load on a single resources factor. Our study thus extends previous research on the hierarchical structuring of psychological concepts, such as intelligence (e.g., Spearman, 1904), personality (e.g., Musek, 2007), or emotionality (e.g., Zinbarg et al., 1997). The resources factor can be interpreted as a fundamental construct comprised of different domains of resources. In other words, this higher-order factor reflects shared variance among different aspects of both personal and job resources. Thus, an individual scoring high in this resources factor is expected to have both high job and high personal resources. In our study, we focused on resources expected to be important in predicting demands, mental strain, and health problems within a working context. Future studies may draw on our findings and test such a resources factor in other research areas as well, by using alternative types of resources relevant for this context.

Although we assumed different mechanisms of action to underly both personal and job resources, we expected the resources factor to be a good predictor of psychosocial job demands, mental strain, and health problems. In fact, we found that high resources were related to a lesser burden due to psychosocial job demands. One explanation for this might be that a person high in resources (vs. a person low in resources) perceives his/her working environment as less demanding due to the belief of being able to cope with the demands and their consequences (Lazarus and Folkman, 1984). The extent of resources available may thus affect the perception of what is stressful and what is not. The effect of resources on psychosocial job demands was relatively strong, and in comparison to previous studies relating job resources to job demands (i.e., Schaufeli and Bakker, 2004; Xanthopoulou et al., 2007; Korunka et al., 2009; Hu et al., 2011), our model ensured a better prediction of job demands. We also found that persons high in resources indicated lower levels of mental strain and of health problems. These results now confirm previous studies which showed that both personal resources and job resources are a beneficial factor for mental and somatic health (Karasek, 1990; Freidl et al., 1999; Kalimo et al., 2002; Ensel and Lin, 2004).

Furthermore, underscoring the health-impairment process of the job demands-resources model (Schaufeli and Bakker, 2004; Hakanen et al., 2006), we found mental strain to mediate the relationship between psychosocial job demands and health problems. That is, a greater burden due to psychosocial job demands was related to higher levels of mental strain, which in turn was associated with a greater number of health problems. In comparison to the aforementioned studies, this indirect effect was slightly weaker due to a less strong effect of mental strain on health problems. A potential explanation for this is that we used an alternative operationalization of mental strain. In contrast to previous studies in which mental strain was operationalized by core dimensions of burnout, we used the variables of exhaustion, alienation, and irritation to account for the mental strain construct. Our intention was to test the robustness of the health-impairment process by using an alternative set of variables indicative of mental strain reactions in a working context. Even though the effect of mental strain on health was not as strong as in previous studies, it can be considered as moderately strong. The relationship between psychosocial job demands and mental strain, however, showed a strong effect. We may thus can conclude that the mental strain factor in the job demands-resources model is not limited to burnout only. Mental states, such as alienation or irritation, could also be important in this context.

Even though we found clear evidence for the indirect pathway, it has to be discussed whether the exclusion of the direct pathway from psychosocial job demands to health problems is legitimate. Although we did not find a considerable decline in model fit by eliminating this direct pathway, the effect of psychosocial job demands on health problems was nonetheless significant. Similarly to Korunka et al. (2009), we found this effect to be negative, in that more psychosocial job demands were related to less health problems. By including mental strain as a mediator, the direct effect seemed to be suppressed for statistical reasons and even changed to a negative effect. However, in comparison to the other coefficients in our model, this effect remained rather weak. Due to the small magnitude of the effect and in terms of the model fit comparisons, we decided to exclude this pathway. Nevertheless, future studies examining the job demands-resources model should take a potential direct pathway and its negative or positive sign into account, in order to clarify our findings.

### 4.1. Strength and limitations

One strength of our model is that it performed well in predicting the dependent variables. The resources factor was able to explain 46% of the variation in the perceived burden due to psychosocial job demands. In mental strain, 84% of the variance was ascribed to psychosocial job demands and the resources factor. Overall, the model explained 44% of the variation in health problems. A further strength of our model is that the combination of personal and job resources into one common factor makes the model more parsimonious and also overcomes statistical issues due to a potentially high correlation between job resources and personal resources. Due to this high correlation, different types of personal and job resources may contain redundant information regarding the prediction of a relevant construct and, as a consequence, meaningful relationships may be falsely obscured.

There are also a number of methodological issues to discuss. First, one weakness of the current research concerns its cross-sectional nature. To reflect our main hypotheses, we applied a confirmatory approach by testing an a priori defined model (Jöreskog and Sörbom, 1993, p. 115). However, our research design only allowed for the interpretation of associations, and no claims can be made about causality. Second, this study may be influenced by common method variance. One potential bias in our findings might be due to the fact that the self-reported data of predictor variables and criterion variables originated from the same person and were collected at the same time. Thus, there may be an artifactual covariance between the predictor and criterion variables, that might have led to biased estimates concerning the relationships between the variables in the model, due to socially desirable responding, the propensity to maintain consistency in responding, or mood states (for further sources of bias, see Podsakoff et al., 2003). Hence, measures of objective data (e.g., physical fitness tests, medical examinations) might have led to more reliable results.

The third limitation concerns the fact that some of the used measures were not validated in previous studies. Instead, we had to select items as proxy measures reflecting the concepts important for our study. This may be a source of bias. For example, the items used for measuring valuable aspects in the context of work did not actually measure job resources as such, but rather satisfaction with job resources. Although a high correlation can be expected between these measures, studies using different instruments may not be fully comparable. To partially account for these shortcomings, we tested and respecified (where necessary) the hypothesized measurement models in order to obtain reliable and valid measures. This, in turn, required the use of modification indexes as a statistical tool for detecting specification errors. However, such an approach is only appropriate with substantive reason for respecification (Hoyle and Panter, 1995, p. 172), and if modifications are then validated on a second sample (MacCallum et al., 1992; MacCallum and Austin, 2000; Raykov and Marcoulides, 2012, p. 51). Since both aspects applied to our study, there is no reason to believe that the modifications made were only due to capitalization on chance. On the other hand, Cole et al. (2007) remind us that modifications (in particular the inclusion of residual covariances) are often necessary, as the failure to include justified specifications can distort the meaning of the latent variables extracted, and in turn, estimates regarding the relationships between latent variables in a structural equation model may be biased.

In addition, it may be considered a limitation that we only modeled the energetical process of the job demands-resources model and disregarded the motivational process. Future research should, therefore, aim to address these issues by integrating both the motivational and energetical process in the model, by using both objective and subjective data measured with validated instruments, and by applying a research design that allows interpretation of cause-and-effect relationships.

### 4.2. Conclusions

In conclusion, we replicated previous findings regarding the basic assumptions of the health-impairment process in the framework of the job demands-resources model. A greater burden due to high psychosocial job demands went along with higher levels of mental strain, which in turn were associated with ill health. In addition, both personal and job resources seemed to be beneficial in reducing the perceived burden due to high psychosocial job demands, and to be valuable factors for promoting mental and somatic health.

Our findings have pointed out that interventions aiming to reduce the risk of work-related health problems could take action on three levels. First of all, the primary occupational objective should be to combat overwhelming psychosocial job demands, since these were found to be strongly related to both mental and somatic health problems. Secondly, efforts could be targeted to the reduction of symptoms of mental strain. Examples for fruitful interventions include the learning of cognitive-behavioral strategies or relaxation techniques (Richardson and Rothstein, 2008). Such interventions can help the working person to cope with extensive job demands and thus prevent the negative side-effects of highly demanding working environments. And thirdly, interventions should aim to enlarge resources. This includes both the improvement of labor conditions as well as the strengthening of physical, mental, and social resources of individuals. These actions could, among other things, be targeted at the evaluation of the individual's own capacities and potentials as well as on the resources available at the workplace. A more positive assessment of one's own resources related to the self or the working environment and the belief of being able to cope with a wide range of potential challenges can be an important buffer against extensive demands and maintain individual well-being (Lazarus and Folkman, 1984; Kalimo et al., 2002).

## Author contributions

Substantial contributions to the conception and design of the work: HM, ES, AW, ÉR, WF. Statistical analyses: HM with input from WF and ES. Interpretation of data: HM, ES, AW, ÉR, WF. Drafting the work: HM with input from WF. Critically revising the manuscript: HM, WF, ES, AW, ÉR. All authors read and approved the final version.

### Conflict of interest statement

The authors declare that the research was conducted in the absence of any commercial or financial relationships that could be construed as a potential conflict of interest.

## Abbreviations

JD-C model: Job demands-control model
ER model: Effort-reward model
JD-R model: Job demands-resources model
COR: Conservation of resources
CFA: Confirmatory factor analysis
SEM: Structural equation modeling
TLI: Tucker-Lewis-Index
CFI: Comparative-Fit-Index
RMSEA: Root Mean Square Error of Approximation.
